# Pharmacogenetics implementation in the clinics: information and guidelines for germline variants

**DOI:** 10.20517/cdr.2018.25

**Published:** 2019-03-19

**Authors:** Gladys Olivera, Luis Sendra, María José Herrero, Pablo Berlanga, Pablo Gargallo, Yania Yáñez, Andrea Urtasun, Jaime Font de Mora, Victoria Castel, Adela Cañete, Salvador F. Aliño

**Affiliations:** ^1^Pharmacogenetics Platform, Instituto de Investigación Sanitaria la Fe, Valencia 46026, Spain.; ^2^Department of Pharmacology, University of Valencia, Valencia 46010, Spain.; ^3^Department of Pediatric and Adolescent oncology, Institute Gustave Roussy Center, Villejuif 94800, France.; ^4^Pediatric Oncology Unit, Hospital Universitario y Politécnico la Fe, Valencia 46026, Spain.; ^5^Pediatric Clinical and Translational Research in Cancer, Instituto de Investigación Sanitaria la Fe, Valencia 46026, Spain.

**Keywords:** Polymorphisms, Pharmacogenetic, PharmGKB, guidelines, oncology, clinical implementation

## Abstract

The aim of this work was to supply an overview of the germline Pharmacogenetics that can be already implemented in the oncology clinical practice. An explanation of the three pillars considered necessary for determining which genetic polymorphisms should be used has been provided. These are PharmGKB single nucleotide polymorphism (SNP)-Drug Clinical Annotations with levels of evidence 1 or 2; the genetic information provided in the drug labels by the drug regulatory main agencies (Food and Drug Administration and European Medicines Agency, mainly); and the guidelines elaborated by international expert consortia (mainly Clinical Pharmacogenetics Implementation Consortium and Dutch Pharmacogenetics Working Group). A summary of the relevant SNPs and the recommendations on how to apply their results has also been compiled.

## Introduction

Pharmacogenetics (PGx) is one of the cornerstones of personalized medicine. It aims to foresee, upon the patient’s genetic characteristics, what drug and which dose would offer the highest therapeutic benefit and/or the lowest probability of adverse effects. The most abundant genetic variants influencing PGx are the single nucleotide polymorphisms (SNPs), accounting for approximately 90% of human genome variability^[1,2]^. However, key variants influencing PGx include also genomic insertions, deletions and repeats, and genetic copy number variations, in addition to SNP. The aim of this review is to provide an easy-to-interpret summary of the drug-germline polymorphisms pairs (mainly SNPs) that currently can be used to help in oncologists in the therapeutic decision making.

PGx is related with pharmacology and thus, the molecular knowledge of drugs’ transporters, metabolizers and mechanism of action are required^[1]^. Adverse drug reactions (ADRs) as well as drug efficacy are associated with particular genetic variants of each individual, related to the genes coding for all the components interacting with the drug inside the patients’ body. For this reason, clinical practice must turn into personalized and precision medicine^[2-4]^. However, how to implement PGx in the daily routine is not free of difficulties and physicians do need the support of rigorous and evidence-based information. The axis of this work consists in a review of the available associations and guidelines of drug/germline polymorphisms, with the highest evidence level, that could be applied within the clinical practice in pediatric and adult oncologic patients. Tumor (somatic) genetic variants are out of the scope of this review. The aim is providing clinicians with a helpful tool for therapeutic prescribing, regarding the individual patient, which can be useful even before having tumor tissue analyses available.

Most of the existing PGx information is compiled in PharmGKB^[3]^, a free access database created, curated and managed by the University of Stanford and funded by US National Institutes of Health (NIH/NIGMS). PharmGKB data are under a Creative Commons license. It counts with a group of experts working on the dissemination of knowledge about the impact of human genetic variation on drug responses and on the translation of PGx into clinical practice.

## Sources of information

In www.pharmGKB.org website an extensive and constantly updated compilation of the PGx knowledge can be found^[3]^. It is mainly based on the results of the articles published worldwide, mainly included in PubMed database. In our case, our focus is the subset of data that is ready to be used in the clinical practice, so our references on this database will be the following:

PGx Prescribing Info: this part contains drug dosing guidelines that take into consideration patient genotype, published by the Clinical Pharmacogenetics Implementation Consortium (CPIC)^[5]^, the Royal Dutch Association for the Advancement of Pharmacy - Pharmacogenetics Working Group (DPWG, manually curated by PharmGKB)^[6]^, or other professional society (PRO, manually curated by PharmGKB)^[7]^.

Drug Labels: Regulatory agencies as the US Food and Drug Administration (FDA)^[8]^, the European Medicines Agency (EMA)^[9]^, the Pharmaceuticals and Medical Devices Agency (PMDA) from Japan^[10]^ and Health Canada Santé Canada (HCSC)^[11]^ have included the recommendation of a genetic test prior to the use of many drugs. The drug label indicates if the test is required, recommendable, actionable or simply informative^[3]^.

Clinical Annotations: drug/polymorphism relationships graded with a “Level of Evidence”. This scientific evidence rank is assigned by the PharmGKB experts from1 to 4 (1A, 1B, 2A, 2B, 3 and 4), 1A being the highest. In level 1A annotations, the variant-drug combination is included in a CPIC or a PGx guide approved by the medical society or implemented in a PGRN (Pharmacogenomics Research Network) site or in another important health system. In level 1B annotations, the preponderance of evidence shows an association that replicates in more than one cohort with significant *P*-values, and preferably will have a strong effect size. Level 2 includes variants with moderate evidence. Level 2A marks annotations for variant-drug combinations that qualify for level 2B where the variant is within a very important pharmacogene as defined by PharmGKB, so functional significance is more likely. In Level 2B variant-drug combinations, the association must be replicated but there may be some studies that do not show statistical significance, and/or the effect size may be small. Level 3 and level 4 clinical associations are not strong enough to be used for clinical translation, under our point of view. Level 3 annotation based on a single significant (not yet replicated) study or annotation for a variant-drug combination evaluated in multiple studies but lacking clear evidence of an association; and finally, level 4 annotations are based on a case report, non-significant study or *in vitro*, molecular or functional assay evidence only^[7]^.

## Drugs and guidelines

Guidelines are not always easy to interpret, mainly if the reader is not used to the usual terms and nomenclature in PGx. It is crucial that the recommendations arrive to the prescribing clinicians in a rigorous but at the same time, simple and easy-to-interpret manner, otherwise clinical implementation of PGx would be hardly impossible.

Following with our aim, we focus now on the existing PGx guidelines for chemotherapy treatment and some associated drugs. Table 1 summarizes the content of CPIC and DPWG published documents, being these, and specially the first, the most active and experienced consortia in providing this kind of tools for PGx translation to the clinic. The description of the procedures employed by each consortium to evaluate the evidence and apply recommendations is described in a couple of publications^[12,13]^. Currently, and being aware that these consortia employ different criteria for nomenclature and also for rising recommendations, they are making efforts for harmonization, with a descriptive publication just comparing their statements and showing the differences as a first step^[14]^.

**Table 1:** Summary of the recommendations provided in CPIC and DPWG guidelines for drugs employed in oncology

Drug	Gene	SNP	Reference genotype	Risk genotype	Guideline recommendation
Azathioprine, Mercaptopurine, Thioguanine^#^					CPIC^[17-19]^
	*TPMT*	rs1800462 rs1800584 rs1142345 rs1800460	CC CC TT CC	CG, GG CT, TT TC, CC CT, TT	Any Heterozygote, including 1/*3A (both risk variants located in the same allele): Consider a reduction starting at 30%-80% of the normal starting dose (if normal starting dose is equal or higher than 75 mg/m^2^/day, or equal or higher than 1.5 mg/kg/day in Mercaptopurine, and 2-3 mg/kg/day in Azathioprine). In the case of Thioguanine, start with 50%-80% of normal dose if it is equal or higher than 40-60 mg/m^2^/day. Allow 2-4 weeks to reach steady state after each dose adjustment Any Homozygote, plus multiple heterozygotes in *2, *3B and *4, that is an individual carrying two functional alleles: Consider alternative agents. If not possible, start with a daily dose reduction 10 times and three times a week. Adjust the dose according to the degree of myelosuppression and the specific patterns of the disease. Allow 4-6 weeks to reach a steady state after each dose adjustment For CT follow the same recommendation as for Heterozygotes in TPMT. For TT, follow the same recommendation as for TPMT Homozygotes, except for Thioguanine, where the reduction should be to 25% DPWG^[6]^ Any Heterozygote: Select alternative drug or reduce dose by 50%. Increase dose in response of hematologic monitoring efficacy Homozygotes or combination of two or more Heterozygotes: Select alternative drug or reduce dose by 90%. Increase dose in response of hematologic monitoring efficacy
	*NUDT15*	rs116855232	CC	CT, TT	
Capecitabine, Fluorouracil^#^					CPIC/DPWG^[6,20,21]^ Other variants are included in the DPWG guideline, but they are extremely rare^[6]^
	*DPYD*	rs3918290	CC	CT, TT	CT: Reduce dose by 50%. TT: Change to alternative agents
		rs55886062	AA	AC, CC	AC: Reduce dose by 50%. CC: Change to alternative agents
		rs3918290+ rs55886062	CC + AA	CT, TT + AC, CC	Change to alternative agents
		rs67376798	TT	AT, AA	Reduce dose by 50%
		rs67376798+ rs3918290	TT + CC	AT, AA + CT, TT	Change to alternative agents
		rs67376798+ rs55886062	TT + AA	AT, AA + AC, CC	Change to alternative agents
		rs75017182	GG	CG, CC	Reduce dose by 50%.
		rs75017182+ rs3918290	GG + CC	CG, CC + CT, TT	Change to alternative agents
		rs75017182+ rs55886062	GG + AA	CG, CC + AC, CC	Change to alternative agents
Tegafur					DPWG^[6]^
	*DPYD*	rs3918290	CC	CT, TT	Homozygotes for any Risk genotype or combination of two heterozygotes: select alternative drug. Fluorouracil or capecitabine are not suitable alternatives because both are also metabolized by DPD. Other variants are included in the guideline, but they are extremely rare^[6]^. Tegafur has no longer recommendations from CPIC based on DPYD genotype. This is due to limited evidence regarding the impact of DPYD variants on tegafur toxicity risk
		rs72549303	G/G	G/del, del/del	
		rs72549309	ATGA/ATGA	ATGA/del, del/del	
		rs1801266	GG	AG, AA	
		rs72549306	CC	AC, AA	
		rs80081766+ rs78060119	CC	CT, TT + AC, AA	
		rs55886062	CC	AC, AA	
		rs1801265	AA	AG, GG	
		rs1801268	CC	AC, AA	
Irinotecan					DPWG^[6]^
	*UGT1A1*	rs8175347	(TA)6/(TA)6 (TA)6/(TA)7	(TA)7/(TA)7	Dose > 250 mg/m^2^: reduce initial dose by 30%. Increase dose in response to neutrophil count. Dose ≤ 250 mg/m^2^: no dose adjustment
Ondansetron^#^					CPIC^[21]^
	*CYP2D6*	-	PM, IM, NM	UM: *1/*1xN, *1/*2xN, *2/*2xN	Select alternative drug not predominantly metabolized by CYP2D6 (i.e., granisetron)
Oxycodone					DPWG^[6]^
	*CYP2D6*		NM	PM: two inactive alleles (*3-*8, *11-*16, *19-*21, *38, *40, *42); IM: two decreased-activity alleles (*9, *10, *17, *29, *36, *41) or carrying one active (*1, *2, *33, *35) and one inactive allele, or carrying one decreased-activity allele and one inactive allele	Select alternative drug (not tramadol or codeine) or be alert to insufficient efficacy
				UM: gene duplication in absence of inactive (*3-*8, *11-*16, *19-*21, *38, *40, *42) or decreased-activity (*9, *10, *17, *29, *36, *41) alleles	Select alternative drug (not tramadol or codeine) or be alert to ADEs
Tamoxifen					CPIC^[22]^ More alleles exist, these are the most common^[22]^
	*CYP2D6*	-	-	UM: *1/*1xN, *1/*2xN, *2/*2xN	Avoid moderate and strong CYP2D6 inhibitors. Initiate therapy with recommended standard of care dosing (tamoxifen 20 mg/day)
		-	-	NM: *1/*1, *1/*2, *1/*9, *1/*41, *2/*2	Avoid moderate and strong CYP2D6 inhibitors. Initiate therapy with recommended standard of care dosing (tamoxifen 20 mg/day)
		-	-	NM or IM (controversy remains): *1/*4, *1/*5, *41/*41; *4/*10, *4/*41, *5/*9; *10/*10, *10/*41	Consider hormonal therapy such as an aromatase inhibitor for postmenopausal women or aromatase inhibitor along with ovarian function suppression in premenopausal women. If aromatase inhibitor use is contraindicated, consideration should be given to use a higher but FDA approved tamoxifen dose (40 mg/day). Avoid CYP2D6 strong to weak inhibitors
		-	-	PM: *3/*4, *4/*4, *5/*5, *5/*6	Recommend alternative hormonal therapy such as an aromatase inhibitor for postmenopausal women or aromatase inhibitor along with ovarian function suppression in premenopausal women given that these approaches are superior to tamoxifen regardless of *CYP2D6* genotype and based on knowledge that CYP2D6 poor metabolizers switched from tamoxifen to anastrozole do not have an increased risk of recurrence. Note, higher dose tamoxifen (40 mg/day) increases but does not normalize endoxifen concentrations and can be considered if there are contraindications to aromatase inhibitor therapy
		-	-	PM: see description in oxycodone	DPWG^[6]^ Increased risk for relapse of breast cancer. Consider aromatase inhibitor for postmenopausal women
		-	-	IM: see description in oxycodone	Increased risk for relapse of breast cancer. Avoid concomitant use of CYP2D6 inhibitors. Consider aromatase inhibitor for postmenopausal women
Tramadol	*CYP2D6*			PM: see description in oxycodone	DPWG^[6]^ Select alternative drug (not oxycodone or codeine) or be alert to insufficient effect
				IM: see description in oxycodone	Be alert to decreased efficacy, consider dose increase. If still inadequate, do as PM
				UM: see description in oxycodone	Reduce dose by 30% and be alert to ADEs, or do as PM

A summary of the published guidelines is shown in this table, translating the information to single SNPs when possible. The original guidelines, especially from CPIC, are much more extensive, so this table is only a comprehensive approach, useful for the majority of cases, but deeper details must be consulted in the original publications. #Applicable to pediatrics. Classical asterisks nomenclature: *1 is always considered the reference genotype. TPMT: *2 equivalent to rs1800462; *3A equivalent to *3B+*3C; *3B equivalent to rs1800460; *3C equivalent to rs1142345; *4 equivalent to rs1800584. NUDT15: *3 equivalent to rs116855232. UGT1A1: *28 equivalent to rs8175347 (this is not a real SNP, but a short tandem repeat polymorphism). DPYD at CPIC guideline: IM, one normal function + one no function, or one decreased function, or two decreased function alleles; PM, two no function, or one no function + one decreased function. No function: c.1905+1G>A equivalent to rs3918290 and DPYD*2A; c.1679 T>G equivalent to rs55886062 and DPYD*13; Decreased function: c.2846 A>T equivalent to rs67376798; c.1129-5923 C>G equivalent to rs75017182. CYP2D6: Date of access to CPIC-DPWG-PharmGKB for guidelines: 15 January 2019. ADE: Adverse Drug Event; UM: ultrarapid metabolizer; NM: normal metabolizer; IM: intermediate metabolizer; PM: poor metabolizer; CPIC: Clinical Pharmacogenetics Implementation Consortium; DPWG: Royal Dutch Association for the Advancement of Pharmacy - Pharmacogenetics Working Group; SNP: single nucleotide polymorphism

Other guidelines, elaborated by other professional groups, are commented afterwards in the text. These guidelines have not received the approval or reached a consensus with other societies or consortia, so the robustness of the way they evaluate the evidences^[15]^ is not recognized in the manner that CPIC is.

Again, it is very important to remark that Table 1 aims to provide a rigorous but user-friendly content, and therefore, translation of asterisk or phenotype-way nomenclatures into “rs” SNP nomenclature is given when possible. Also, some of the guidelines include very low frequent variants that are not considered in Table 1 either, so if a deeper detail is needed, the original guidelines referenced in this work should be consulted.

Regarding *CYP2D6*, it is necessary to state that it is one of the most complicated genes to analyze in terms of genotyping and in knowing with high certainty, the genotype-phenotype correlation. Many groups are working on this point worldwide, trying to clearly identify which genetic variants lead to poor, intermediate, normal or ultrarapid metabolizers (UM). This gene is highly polymorphic, but not only regarding SNPs content, but also in duplications, deletions, *etc*. Drug-drug interactions and ethnic variability are very important for every pharmacogene, but we could say that for *CYP2D6* the impact is the highest^[16]^. That is why in Table 1, the information regarding ondansetron, oxycodone, tamoxifen and tramadol has been kept with the typical *CYP2D6* nomenclature including haplotypes and asterisks.

### Thiopurines and TPMT

Thiopurine drugs such as azathioprine (AZA), 6-mercaptopurine (6-MP) and 6-thioguanine, are cytotoxic drugs employed in the treatment of severe diseases as childhood acute lymphoblastic leukemia and inflammatory bowel disease^[23]^. AZA is converted almost completely to 6-MP through a non-enzymatic reaction within the liver^[24]^.

These drugs are administered as prodrugs that are converted to thioguanine nucleotides (TGN) by the hypoxanthine guanine phosphoribosyltransferase enzyme. TGNs integrate within DNA and RNA leading to cancer cell death, what is inactivated by cytosolic Thiopurine S-methyltransferase (TPMT) enzyme via S-methylation. For this reason, *TPMT* gene polymorphisms can alter the enzyme activity and trigger the apoptosis of healthy cells and lead to ADRs^[2]^.

Alleles *2 (rs1800462, C>G), *3A [haplotype *3B (rs1800460, C>T) + *3C (rs1142345, T>C)], *3B, *3C and *4 (rs1800584) are the most common variants and they are estimated to foresee up to 90% of TPMT function and variability. Other 34 *TPMT* alleles with low frequencies in different populations have been also described.

Initial doses of treatment with thiopurines are high since they derive from clinical trials performed in general population, where wild type allele has a frequency of 86%-97%. These standard doses must be administered only in those patients homozygous for wild type *TPMT* gene (*1/*1).

CPIC recommendations indicate that those patients with heterozygous *TPMT* (one functional allele *1 and one non-functional allele), should initiate the treatment with 30%-80% of the target dose and been evaluated according to tolerance. Finally, in those patients homozygous for non-functional variants of *TPMT* it is recommended to begin with 10% of the target dose and 3 doses a week instead of daily treatment or to change the drug employed^[15]^.

The DPWG provides another clinical guide^[6]^ for the use of thiopurines. They recommend in intermediate metabolizers (IM) heterozygous for *TPMT* function (containing 1 functional allele as *1, *1S, *1A, and one non-functional allele like *2, *3A-*3D, *4-*18) to select an alternative drug or reduce the dose to 50% and increase it under efficacy and hematologic surveillance. In poor metabolizers (PM) patients carrying two inactive alleles (*2, *3A-*3D, *4-*18) it is recommended to select an alternative drug or to reduce the doses by 90% and increase it according to the efficacy and hematologic toxicity data obtained by monitoring the patient.

However, there are patients with *TPMT* wild type genotype that present toxicity when treated with thiopurines. This is most probably due to the existence of other variants involved in thiopurines metabolism such as the observed in a GWAS study that correlated the variant rs116855232 C>T in *NUDT15* gene^[25]^ with myelosuppression in inflammatory bowel disease and acute lymphoblastic leukemia patients treated with thiopurines^[26]^. Different studies correlate the CT or TT genotype with higher toxicity risk and conclude that doses should be reduced^[27]^.

### Capecitabine, fluorouracil, tegafur and DPYD

Fluoropyrimidines, capecitabine, fluorouracil and tegafur, are antimetabolite drugs widely employed in colorectal, aerodigestive tract and breast cancer treatment. Between 10% and 40% of the patients with this type of treatment develop severe toxicity (neutropenia, nausea, vomit, diarrhea, stomatitis, mucositis, foot hand syndrome), causing even the death in some cases^[28]^.

The most common cause of fluoropyrimidines toxicity is the lack of the key enzyme for fluorouracil metabolism, dihydropyrimidine dehydrogenase (DPD), which is encoded by *DPYD* gene. A complete lack of enzyme activity is only present in very few patients. Most patients have a reduced DPD activity due to the genetic risk variant, but not a complete lack of activity since most patients are heterozygous carriers of these variants and thus have one fully functional gene copy. Reduced activity is present in 39%-61% of patients with severe toxicity, what highlights its relevance as severe toxicity risk factor^[29]^.

Nowadays, different *DPYD* gene variants resulting in reduced enzyme function and toxicity risk are known and described. Variants rs3918290 (G>A) or *2A and rs55886062 (T> G) or *13 present higher impact in reducing enzyme activity than variants rs67376798 (A>T) and rs75017182(C>G), which are associated with moderately reduced enzyme activity.

According to the CPIC guide^[20]^, the dosing recommendations for fluoropyrimidines are based on individuals genotype, which can be divided in three main groups: normal metabolizers (NM), carrying two normal alleles; IM carrying one normal allele and another one with reduced function, or carrying two alleles with reduced function; and PM, carrying two alleles without function or one non-functional allele and another one with reduced function.

In NM the administration and doses indicated in drug label are recommended. In intermediate metabolizers, it is recommended to reduce the doses by 50%. Finally, the poor metabolizers are recommended to be treated with therapeutic schemes without 5-fluorouracil.

PharmGKB explains the Drug Agencies information in the drug label indicating that “actionable”, means that “the label does not discuss genetic or other testing for gene/protein/chromosomal variants, but does contain information about changes in efficacy, dosage or toxicity due to such variants. The label may mention contraindication of the drug in a particular subset of patients but does not require or recommend gene, protein or chromosomal testing”. Labels approved by FDA, EMA, HCSC and PMDA for capecitabine point that those patients with low or absent DPD activity present higher risk of severe or lethal adverse reactions. Fluorouracil does not appear within EMA label.

There is still scarce information about the *DPYD* gene variants in pediatric patients. However, there is no evidence that these variants could affect the 5-fluorouracil metabolism in children in a different way to adults.

The DPWG guide for capecitabine and fluorouracil recommend to reduce the dose by 50% or to change the drug in intermediate metabolizers. For poor metabolizers it is recommended to use an alternative drug too. Tegafur is not an appropriate alternative since it is also a substrate for DPD.

Tegafur is another fluorpyrimidine that does not present a CPIC recommendation. However, the DPWG guide has a recommendation to poor metabolizers to select an alternative drug, avoiding capecitabine and fluorouracils since they are also metabolized by DPD^[6]^.

### Irinotecan and UGT1A1

Irinotecan is a hemisynthetic camptothecin mainly used for the treatment of metastatic colorectal cancer, multiform glioblastoma, lung cancer, upper gastrointestinal cancer and pancreas cancer. It is metabolized by the liver to be converted in its active metabolite called SN-38. The SN-38 targets DNA topoisomerase I, stabilizing the cleavable complexes that break the DNA strands and producing the cancerous cells death^[30,31]^.

The *UGT1A1* (UDP Glucuronosyltransferase family 1 member A1) enzyme is the responsible of SN-38 inactivation and detoxification. Variants of this gene, such as *28, related with reduced activity of this enzyme, increase the blood levels of SN-38 metabolite and its toxicity^[32]^.

The DPWG guide recommends in patients with a programmed dose of more than 250 mg/m^2^, to reduce the initial dose of irinotecan by 30% in homozygous (*28/*28) poor metabolizer patients, and increase it as response to neutrophils count^[6]^. UGT1A1*28 has an rs code, rs8175347, but it is not a SNP, instead is a variable number tandem repeat of a dinucleotide TA. There is a surrogate marker, according to the CPIC Atazanavir guideline^[33]^, rs887829 that could be an alternative to analyze this variant.

The French National Network of Pharmacogenetics (RNPGx) and the Group of Clinical Oncopharmacology (GPCO-Unicancer) also presented a guide for *28 allele^[34]^. When initial doses of irinotecan are between 180 and 230 mg/m^2^ every 2-3 weeks homozygous *28/*28 patients present greater risk of hematologic and digestive toxicity than other genotypes. This guide recommends in this case to reduce the initial doses by 25%-30%, especially in those patients with associated risk factors.

When the initial dose is ≥ 240 mg/m^2^ every 2-3 weeks, the neutropenia risk is greater. This dose is contraindicated for *28/*28 homozygous patients. It would be possible only in patients homozygous for wild type allele or heterozygous *1/*28 without other associated risk factors and under rigorous surveillance (the same in 180-230 mg/m^2^).

FDA and HCSC agencies include recommendations within the drug label, considered actionable. They indicate that the initial dose in *28 homozygous patients should be considered to be reduced because of the hematologic toxicity risk. It is also recommended not to use neither CYP3A4 inducers during two weeks before initiating irinotecan treatment nor strong CYP3A4 inhibitors one week before and during the irinotecan treatment period. PMDA agency indicates the requirement of genetic test prior to drug administration.

### Ondansetron and CYP2D6

Ondansetron is a serotonin receptor antagonist (5-hydroxitriptamine, subtype 3), employed as antiemetic after chemotherapeutic or surgical treatment. It is metabolized within the liver by cytochrome P450 enzymes, specifically CYP2D6. This gene presents large amount of variants that can mediate higher or lower efficacy and toxicity^[35-37]^.

The CPIC guide presents recommendations for this drug. It assigns an activity value from 0 to 1 to each functional group. In this sense, this activity value is 0 for poor metabolizers, 0.5 for intermediate metabolizers and 1 for NM. When the allele has more than 1 copy of the functional gene, the activity value is multiplied by the copy number present. Thus, the global activity value is obtained by adding the values of each allele. The patients with an activity value over 2 will be considered UM^[38]^.

The recommendations depend, then, upon the group to which each patient belongs. Nowadays, only those patients included within the UM (allelic combinations *1/*1xN, *1/*2xN, *2/*2xN) group have recommendations. This guide recommends using an alternative drug not metabolized by CYP2D6. The authors state that there is no reason to expect differences in pediatric oncology, except in newborns^[21]^.

The recommendations are considered informative by FDA.

### Tamoxifen and CYP2D6

Around 65%-75% of breast cancers express estrogen receptors (ER) or progesterone receptors. This type of cancer can be treated with endocrine therapy such as tamoxifen since it is a selective modulator of estrogen receptor.

Tamoxifen is the unique hormonal agent approved by FDA for pre-menopausal breast cancer prevention, *in* *situ* ductal carcinoma treatment and as adjuvant treatment in invasive pre-menopausal metastatic breast cancer.

The metabolization of tamoxifen takes place within the liver and is performed by cytochrome P450 enzymes. The main metabolism mechanism contributes to up to 90% of the global tamoxifen metabolism and consists in the demethylation of tamoxifen to N-demethyltamoxifen by CYP3A4^[39]^, followed by the oxidation to 4-hydroxi-N-demethylmetamoxifen (endoxifen) mediated by CYP2D6^[40]^.

The gene variants are described in https://cpicpgx.org/alleles/^[41]^.

Both CPIC and DPWG have guides for the use of tamoxifen. CPIC guide focuses on the CYP2D6 role in the ER+ cancer breast adjuvant treatment. The recommendations for ultrarapid (*1/*1xN, *1/*2xN, *2/*2xN) and normal (*1/*1, *1/*2, *1/*9, *1/*41, *2/*2) metabolizers are to avoid strong and moderate CYP2D6 inhibitors. Treatment can initiate with the standard tamoxifen dose (20 mg/day).

In intermediate metabolizers (*4/*10, *4/*41, *5/*9, *1/*4, *1/*5, *41/*41, *10/*10, *10/*41), aromatase inhibitor therapy should be considered in post-menopausal patients and aromatase inhibitor combined with ovary function suppression in pre-menopausal women since these methods present better outcomes than tamoxifen regardless *CYP2D6* genotype^[42]^. In those cases the aromatase inhibitors are contraindicated, tamoxifen doses higher than those approved by the FDA (40 mg/day) should be considered. It must be taken into account that highest dose of tamoxifen (40 mg/day) increases the concentration of endoxifen without reaching normal levels and then it could be considered if aromatase inhibitors contraindications exist^[43,44]^.

Lastly, in poor metabolizer patients (*3/*4, *4/*4, *5/*5, *5/*6) it is recommended to use an alternative hormonal therapy such as aromatase inhibitor in post-menopausal patients and an aromatase inhibitor combined with ovary function suppression in pre-menopausal women because these strategies are more efficient than tamoxifen regardless the *CYP2D6* genotype especially if they are deficient for CYP2D6 metabolism. Changing tamoxifen by anastrozole does not present higher recurrence risk.

The DPWG also elaborated a guide of recommendation directed to poor metabolizer genotypes (*3-*8, *11-*16, *19-*21, *38, *40, *42) and intermediate metabolizer genotypes [two reduced activity alleles (*9, *10, *17, *29, *36, *41) or one active allele (*1, *2, *33, *35) and one inactive (*3-*8, *11-*16, *19-*21, *38, *40, *42) or one inactive allele (*3-*8, *11-*16, *19-*21, *38, *40, *42) and one reduced activity allele (*9, *10, *17, *29, *36, *41) and one inactive (*3-*8, *11-*16, *19-*21, *38, *40, *42) allele]. It is recommended the use of aromatase inhibitors in post-menopausal women since higher risk of relapse in breast cancer exists. Also, the concomitant use of CYP2D6 inhibitors must be avoided for intermediate metabolizers^[6]^.

### Guidelines from other professional societies

Cisplatin is one of the most efficacious chemotherapeutic agent in pediatrics, widely used in the treatment of diverse solid tumors such as neuroblastoma, hepatoblastoma, brain tumors, osteosarcoma and germ cell tumors^[45]^. One of the most important complications of this drug is its risk of ototoxicity that produces permanent bilateral audition loss in 26% to 90% of the children treated and 10%-25% of adults^[46]^.

There are several studies that suggest that genetic factors may be involved in ototoxicity, although the results are contradictory and scarce in children^[47]^. Studies of gene variants are related to the cytotoxic effect of cisplatin, where genes such as *GSTP1, SOD2* (rs4880), *XPC* (rs2228001), *XPD* (rs1799793), or genes related to transport are involved such as *SLC* family (rs4788863)^[48-52]^.

Currently there are no CPIC nor DPWG guidelines for this drug. However, CPNDS has published a guide relating cisplatin ototoxicity with *TPMT* variants^[15]^, even if the works they refer to are controversial^[53-56]^.

The CPNDS elaborated a guide with recommendations based on the levels of classification for clinical practice. Level A corresponds to high level of evidence (benefits clearly overcome the risks); level B is a recommendation with lower scientific evidence level based on expert opinion; and level C is mainly based in experts opinion to be used in research context. This guide recommends with level A to perform pharmacogenetics tests in pediatric patients with variants *2, *3A, *3B and *3C related with reduced enzyme activity and audition loss^[54,55,57-59]^. There are recommendations with evidence level C such as the consideration to use otoprotectors in those patients with non-functional variants. Also, the prescription of an alternative treatment when presenting the same efficacy, low toxicity and less ototoxicity^[60,61]^. More frequent monitoring and tracing audiometric control after treatment are also recommended. The impact of these variants related with audition loss is unknown in adult patients^[62,63]^.

There is also a document for doxorubicin. Anthracyclines, as doxorubicin, are among the most effective anticancer treatments, with survival rates over 80% in some kinds of cancer^[64]^. They are employed in the treatment of children and adults’ leukemia, lymphoma and some solid tumors such as breast cancer, ovarian cancer, lung cancer and sarcomas. They act by blocking the synthesis of DNA and RNA by inhibiting topoisomerase II enzyme, thus interrupting DNA replication and transcription and, hence, the replication of cancer cells. Anthracyclines also damage the DNA, proteins and membranes of rapid division cells by creating free oxygen radicals mediated by iron^[65,66]^.

The clinical use of anthracyclines is mainly limited by the high inter-individual variability in anthracycline-induced cardiotoxicity (ACT) which is dependent on the cumulative dose of the drug and produces toxic effects on heart muscles and their conductivity. ACT occurs in 57% of patients treated and is still an important limitation of chemotherapy based on anthracyclines^[67,68]^.

There is a guide for doxorubicin elaborated by the CPNDS^[69]^ in which it is recommended to perform a pharmacogenetic test for the variants rs2229774 (G> A) in the gene *RARG* and rs17863783 (G> T) in the UGT1A6*4, considered of high risk for developing ACT and the variant rs7853758 in the *SLC28A3* gene whose allele A is associated with a reduced risk of ACT^[68,70]^. This allows a stratification of the patients: low-risk patients are recommended a routine echocardiogram and according to the long-term follow-up guidelines of the Children’s Oncology Group (COG), a cardiac follow-up every 5 years^[71]^. For patients at moderate risk, an increase in the frequency of echocardiograms is recommended, such as the monitoring of cardiotoxicity and, according to the COG, a follow-up every 2 years^[71,72]^. Finally, for high-risk patients, the following recommendations should be considered: the increase in the frequency of annual echocardiograms and monitoring before each administration of anthracyclines as well as the follow-up recommended by the COG guidelines^[71]^; as well as the “aggressive” detection and the examination of risk factors such as cardiovascular, such as obesity, diabetes, hypertension, coronary artery disease, lipid disorders and peripheral vascular disease^[73]^.

While Table 1 shows the drug-SNP pairs that are currently included in a guideline, Table 2 shows a compilation of the current information according to the three main pillars explained at the initial section of this work. It includes the majority of drugs used in chemotherapy treatments, and some antiemetic and pain relief drugs that reach Clinical Annotations with Levels of Evidence 1 and 2, listed in PharmGKB, but still do not have a therapeutic/dosing guideline published. Some SNPs may not be directly described in PharmGKB Clinical Annotations under a drug name, but instead they can be found under the generic name of that drug’s family. For instance, some relevant SNPs assigned to “cisplatin”, can only be reached by searching “platinum compounds”. The possible genotypes for each SNP are divided into “Reference Genotype” and “Risk Genotype”, having defined these groups in agreement with the biological or clinical meaning of each genotype. Not necessarily the positive or negative effects of a given genotype are linked to the most or least frequent variants, respectively. Variant frequencies can vary widely between different ethnical human populations.

**Table 2:** Summary of SNP-drug relevant associations according to PharmGKB and drug labels

Drug	Gene	SNP	Reference Genotype	Risk Genotype	CA Level: D, E, T, Pk	Drug label	Implications
Azathioprine	*ITPA*	rs7270101	AA	AC, CC	2B: T	T	Moderate risk of toxicity
Capecitabine	*UMPS*	rs1801019	GG, CG	CC	2B: T	A	Moderate risk of toxicity
Carboplatin	*ERCC1*	rs11615	GG	AA, AG	2B: E, T	I	Moderate risk of inefficacy and toxicity
	*ERCC1*	rs3212986	AA	AC, CC	2B: T		Moderate risk of toxicity
	*GSTP1*	rs1695	GG	AA, AG	2A: T		Moderate risk of toxicity
	*MTHFR*	rs1801133	AA	AG, GG	2A: E		Moderate risk of inefficacy
	*NQO1*	rs1800566	GG	AA, AG	2A: E		Moderate risk of inefficacy
	*XRCC1*	rs25487	CC	CT, TT	2B: E		Moderate risk of inefficacy
Cyclophosphamide	*GSTP1*	rs1695	AA, AG	GG	2A: E, T	I	Moderate risk of inefficacy and toxicity
	*SOD2*	rs4880	AA	AG, GG	2B: E		Moderate risk of inefficacy
	*TP53*	rs1042522	CC	CG, GG	2B: E, T		Moderate risk of inefficacy and toxicity
Cisplatin	*ERCC1*	rs11615	GG	AA, AG	2B: E, T	I	Moderate risk of inefficacy and toxicity
	*ERCC1*	rs3212986	AA	AC, CC	2B: T		Moderate risk of toxicity
	*GSTP1*	rs1695	AA	AG,GG	2B: T		Moderate risk of toxicity
	*NQO1*	rs1800566	GG	AA, AG	2A: E		Moderate risk of inefficacy
	*TP53*	rs1042522	CC	CG, GG	2B: E, T		Moderate risk of inefficacy and toxicity
	*XPC*	rs2228001	TT	GT, GG	1B: T		High risk of toxicity
	*XRCC1*	rs25487	CC	CT, TT	2B: E		Moderate risk of inefficacy
Doxorubicin	*NQO1*	rs1800566	GG	AA, AG	2A: E	I	Moderate risk of inefficacy
Etoposide	*DYNC2H1*	rs716274	AA	AG, GG	2B: T	I	Moderate risk of toxicity
Fentanyl,Metadone, Morphine,Opioids, Oxycodone, Tramadol	*ABCB1*	rs1045642	AA, AG	GG	2B: D, E	*	Moderate risk of inefficacy. Consider dose increase
Fluorouracil	*GSTP1*	rs1695	AG, GG	AA	2A: E	A	Moderate risk of inefficacy
	*TP53*	rs1042522	CC	CG, GG	2B: E, T		Moderate risk of inefficacy and toxicity
	*UMPS*	rs1801019	GG, CG	CC	2B: T		Moderate risk of toxicity
Irinotecan	*C8ORF34*	rs1517114	GG	CG, CC	2B: T	A	Moderate risk of toxicity
	*SEMA3C*	rs7779029	TT	CT, CC	2B: T		Moderate risk of toxicity
	*UGT1A1*	rs4148323	GG	GA, AA	2A		Moderate risk of toxicity
Methotrexate	*ABCB1*	rs1045642	GG	AG, AA	2A: T	I	Moderate risk of toxicity
	*ATIC*	rs4673993	CC, TC	TT	2B: E		Moderate risk of inefficacy
	*MTHFR*	rs1801133	GG	AA, AG	2A: D, E, T		Consider dose reduction
	*MTRR*	rs1801394	AA	AG, GG	2B: T		Moderate risk of toxicity
	*SLCO1B1*	rs11045879	CC	CT, TT	2A: T		Moderate risk of toxicity
Ondansetron	*ABCB1*	rs1045642	AA	AG, GG	2A: E	I	Moderate risk of inefficacy
Oxaliplatin	*ERCC1*	rs11615	GG	AA, AG	2B: E, T	I	Moderate risk of inefficacy and toxicity
	*ERCC1*	rs3212986	AA	AC, CC	2B: T		Moderate risk of toxicity
	*GSTP1*	rs1695	GG	AA, AG	2A: T		Moderate risk of toxicity
	*NQO1*	rs1800566	GG	AA, AG	2A: E		Moderate risk of inefficacy
	*XRCC1*	rs25487	CC	CT, TT	2B: E		Moderate risk of inefficacy
Paclitaxel	*TP53*	rs1042522	CC	CG, GG	2B: E, T	I	Moderate risk of inefficacy and toxicity
Rituximab	*FCGR3A*	rs396991	CC, AC	AA	2B: E	T	Moderate risk of inefficacy
Tamoxifen	*CYP2D6*	rs3892097	CC, CT	TT	2A: E	T	Moderate risk of inefficacy
	*CYP2D6*	rs3892097	TT	CT, CC	2A: T		Moderate risk of toxicity
Tegafur	*DPYD*	rs67376798	TT	AA, AT	1A: T, Pk	I	High risk of toxicity
	*UMPS*	rs1801019	GG, CG	CC	2B: T		Moderate risk of toxicity
Trastuzumab	*FCGR2A*	rs1801274	AA	AG, GG	2B: E	T	Moderate risk of inefficacy
	*FCGR3A*	rs396991	CC	AC, AA	2B: E		Moderate risk of inefficacy
Vincristine	*CEP72*	rs924607	CC, CT	TT	2B: T	I	Moderate risk of toxicity

*As this line describes a group of drugs, no information from a single drug label can be provided. This table lists the SNPs with levels of evidence 1 and 2 for the chemotherapeutic drugs and some others frequently employed in cancer patients (ondansetron and some pain relief drugs), that still have not a published guideline with therapeutic recommendations. The information about the “reference” and “risk” genotypes, with the proposed implications if the patient bears the risk one is provided. “CA” column describes the level of evidence and the way in which the risk genotype affects the drug mechanism (D, dosage; E, efficacy; Pk, pharmacokinetics; T, toxicity) and what does the drug label (FDA or EMA) state about performing genetic tests when prescribing that drug (T, testing required or recommended; A, actionable; I, informative), not specifying genes or variants. SNPs related with a set of drugs have not been listed, this table only includes the genes and SNPs directly linked with each individual drug. Date of PharmGKB access 15 January 2019. SNPs: single nucleotide polymorphisms; CA: clinical annotation

The “Implications” column provides an easy-to-use short interpretation of the content in the original resource. The last column of the table shows the existence of a genetic test recommendation in the drug label, according to FDA and/or EMA.

## Conclusion

This work aims to summarize and make available to clinicians, in a practical way, the guidelines and recommendations that exist so far with high level of evidence, of germline genetic variants associated with drugs used in the treatment for both pediatric and adult oncology. In this way, our aim is to facilitate the comprehension of the available information for decision making in clinical practice. The big consortia, as well as relevant hospitals’ initiatives worldwide, are performing strong efforts to harmonize, to disseminate and to integrate PGx as a real and useful tool for the clinicians’ routine^[74]^.

PGx aims to improve patient’s life quality, reducing the adverse effects of drugs and improving the effectiveness of these. Approximately 20% of pediatric oncology patients do not respond to standard treatments, this percentage seems to be even higher in adults, where the available data on the risks and benefits of treatment are scarce. Administered chemotherapeutic schemes include high doses and toxicity risk, leading to a wide variety of side effects. This is often due to the low specificity of therapeutic target and to the treatment intensification. Chemotherapy toxicity is a regular cause of morbidity and mortality in most of these patients, with short- and long-term sequels. At present, there are PGx tests with sufficient evidence to be implemented in clinical practice, regulated by different guides and drug regulatory agencies. So far, most of them are aimed for treatment in adults and only well defined for its use in pediatric oncology in a few cases. Under our point of view, CPIC guidelines are currently the ones that reflect the most updated information, evaluating a larger quantity of published data, and reaching more international experts’ consensus.

There are still many variants associated with drugs that have not reached sufficient evidence to be implemented in clinical practice due to the lack of consensus in the studies or the disparity of results. For this reason, PGx studies should continue to be carried out in order to reinforce those variants well described and contribute to increase the evidence in those for which, at the moment, the evidence is not high enough to consider their inclusion in clinical practice.

## Declarations

### Copyright

© The Author(s) 2019.
